# Biglycan- and Sphingosine Kinase-1 Signaling Crosstalk Regulates the Synthesis of Macrophage Chemoattractants

**DOI:** 10.3390/ijms18030595

**Published:** 2017-03-09

**Authors:** Louise Tzung-Harn Hsieh, Madalina-Viviana Nastase, Heiko Roedig, Jinyang Zeng-Brouwers, Chiara Poluzzi, Stephanie Schwalm, Christian Fork, Claudia Tredup, Ralf P. Brandes, Malgorzata Wygrecka, Andrea Huwiler, Josef Pfeilschifter, Liliana Schaefer

**Affiliations:** 1 Pharmazentrum Frankfurt, Institut für Allgemeine Pharmakologie und Toxikologie, Klinikum der Goethe Universität, Theodor-Stern-Kai 7, Frankfurt am Main 60590, Germany; hsiehlouise@gmail.com (L.T.-H.H.); madalina.nastase@gmail.com (M.-V.N.); heiko.roedig@live.de (H.R.); jinyangzeng@web.de (J.Z.-B.); chiarapoluzzi@yahoo.it (C.P.); s.schwalm@med.uni-frankfurt.de (S.S.); tredup@em.uni-frankfurt.de (C.T.); pfeilschifter@em.uni-frankfurt.de (J.P.); 2 Institut für Kardiovaskulare Physiologie, Klinikum der Goethe-Universität, Theodor-Stern-Kai 7, Frankfurt am Main 60590, Germany; fork@vrc.uni-frankfurt.de (C.F.); brandes@vrc.uni-frankfurt.de (R.P.B.); 3 Department of Biochemistry, Faculty of Medicine, Universities of Giessen and Marburg Lung Center, Friedrichstrasse 24, Giessen 35392, Germany; malgorzata.wygrecka@innere.med.uni-giessen.de; 4 Institute of Pharmacology, University of Bern, Inselspital INO-F, Bern CH-3010, Switzerland; huwiler@pki.unibe.ch; 5 National Institute for Chemical-Pharmaceutical Research and Development, 112 Vitan Avenue, Bucharest 031299, Romania; madalina.nastase@gmail.com

**Keywords:** damage-associated molecular pattern, small leucine-rich proteoglycan, toll-like receptors, chemoattractant, extracellular matrix, lipid signaling, sphingolipid, macrophage

## Abstract

In its soluble form, the extracellular matrix proteoglycan biglycan triggers the synthesis of the macrophage chemoattractants, chemokine (C-C motif) ligand CCL2 and CCL5 through selective utilization of Toll-like receptors (TLRs) and their adaptor molecules. However, the respective downstream signaling events resulting in biglycan-induced CCL2 and CCL5 production have not yet been defined. Here, we show that biglycan stimulates the production and activation of sphingosine kinase 1 (SphK1) in a TLR4- and Toll/interleukin (IL)-1R domain-containing adaptor inducing interferon (IFN)-β (TRIF)-dependent manner in murine primary macrophages. We provide genetic and pharmacological proof that SphK1 is a crucial downstream mediator of biglycan-triggered CCL2 and CCL5 mRNA and protein expression. This is selectively driven by biglycan/SphK1-dependent phosphorylation of the nuclear factor NF-κB p65 subunit, extracellular signal-regulated kinase (Erk)1/2 and p38 mitogen-activated protein kinases. Importantly, in vivo overexpression of soluble biglycan causes Sphk1-dependent enhancement of renal CCL2 and CCL5 and macrophage recruitment into the kidney. Our findings describe the crosstalk between biglycan- and SphK1-driven extracellular matrix- and lipid-signaling. Thus, SphK1 may represent a new target for therapeutic intervention in biglycan-evoked inflammatory conditions.

## 1. Introduction

Inflammation can be triggered by various external microbial stimuli as well as by endogenous molecules under sterile conditions. The latter are called damage-associated molecular patterns (DAMPs) and are released following cell death or tissue injury [1].

Biglycan, a small leucine-rich proteoglycan (SLRP) of the extracellular matrix (ECM) [2] is normally sequestered in the ECM and therefore inactive as inflammatory trigger [3,4]. However, following tissue stress, biglycan is proteolytically released from the ECM and in its soluble form acts as a DAMP, thereby initiating a sterile inflammatory response [5]. Besides its liberation from the ECM, biglycan can also be synthesized de novo by macrophages and resident cells in inflamed tissues [5,6,7]. Soluble biglycan is a high-affinity ligand of the Toll-like receptors (TLR)2 and TLR4, mimicking the response to gram-positive (via TLR2) and gram-negative (via TLR4) pathogens [5,6]. By binding to TLR2/4 with downstream activation of the extracellular signal-regulated kinase (Erk)1/2 and p38 mitogen-activated protein kinases (MAPKs) as well as the nuclear factor κ-light-chain-enhancer of activated B-cells (NF-κB) pathway, biglycan triggers the synthesis of tumor necrosis factor (TNF)α and interleukin-1 (IL-1)β cytokines [5,8,9].

There are two adapter molecules essential for the TLR signaling: the myeloid differentiation primary response protein (MyD88) and Toll/IL-1R domain-containing adaptor inducing interferon (IFN)-β (TRIF). While signaling through TLR4 involves both MyD88 and TRIF adapters the TLR2 signaling pathway requires exclusively MyD88 for NF-κB activation [10]. It is well documented that the biglycan protein core is solely responsible for the high affinity binding of this proteoglycan to TLR2 and TLR4 [11]. On the other hand, only fully glycanated intact biglycan, consisting of the protein core and two glycosaminoglycan side chains, is capable of inducing TLR2 and TLR4 signaling [5,8]. The structural motifs of biglycan protein and the adapter molecules involved in these interactions need further investigations. By selective engagement of TLRs and their adaptor molecules biglycan tightly regulates inflammatory outcome [6,7,11,12]. Accordingly, biglycan-induced recruitment of macrophages to the kidney depends on biglycan-triggered transcription and secretion of macrophage chemoattractants, chemokine (C-C motif) ligand CCL2 and CCL5 [4,5,6,9,13,14]. Previously, we showed that circulating biglycan evokes the production of CCL2 in a TLR2/4/MyD88-dependent manner, whereas the production of CCL5 was TLR4/TRIF dependent [6]. The interactions between biglycan and different receptors [3,4] orchestrate the recruitment of macrophages to inflamed tissues under disease conditions such as in lupus nephritis [9] and renal ischemia-reperfusion injury [11,15]. However, to date, the exact molecular mechanism through which biglycan-induced TLR2/TLR4/MyD88 and TLR4/TRIF pathways lead to the production of CCL2 and CCL5 remain elusive.

There is growing evidence that sphingolipid signaling plays an essential role in the modulation of various inflammatory pathways [16]. Sphingosine kinases (SphK)s, with the two isoforms SphK1 and -2, are enzymes, which catalyze the adenosine triphosphate (ATP)-dependent phosphorylation of sphingosine (Sph) to produce sphingosine 1-phosphate (S1P) [17,18]. S1P is implicated in cellular processes such as cell survival, proliferation, differentiation, migration, and immune function [18]. Given these roles of S1P, the sphingosine kinases activity is a target in many pathological conditions such as atherosclerosis, acute pulmonary injury, respiratory distress, tumorigenesis, and metastasis as well as inflammation [19].

In response to TNFα or IL-1β, SphK1 is phosphorylated by Erk1/2, which increases its catalytic activity [20]. Furthermore, SphK1 and the production of S1P increase the activity of the TNF receptor-associated factor 2 (TRAF2) E3 ubiquitin ligase, receptor-interacting protein 1 (RIP1) polyubiquitination and further NF-κB activation [21]. This potentiates the expression of chemokine (C-X-C motif) ligand (CXCL) 10 and CCL5 resulting in the recruitment of the mononuclear cells to the site of inflammation [22]. Moreover, lipopolysaccharides (LPS) induce SphK1 activation via TLR4 in macrophages, thereby promoting IL-6 generation [23]. Targeting SphK1 in mice by genetic ablation or pharmacological inhibition ameliorates the inflammatory cytokine production as well as the pathogenesis of experimental models of arthritis [24,25], hepatitis [26], and pulmonary fibrosis [27]. Based on these reports, it is tempting to speculate that there is a reciprocal interference between biglycan and sphingolipid signaling in the regulation of inflammation.

Here we demonstrate for the first time that there is crosstalk between the ECM-derived component biglycan and SphK1-driven lipid signaling. We show that soluble biglycan enhances the expression and activity of SphK1 via the TLR4/TRIF pathway in mouse primary macrophages. Biglycan-induced SphK1 activity is essential for the production of CCL2 and CCL5 chemoattractants. Importantly, we prove this concept in vivo in soluble biglycan-overexpressing mice deficient for SphK1. Thus, targeting SphK1 may represent a potential therapeutic strategy in biglycan-evoked sterile inflammation.

## 2. Results

### 2.1. Biglycan Triggers the Expression and Activity of Sphk1 in Mouse Peritoneal Macrophages

**Figure 1:**
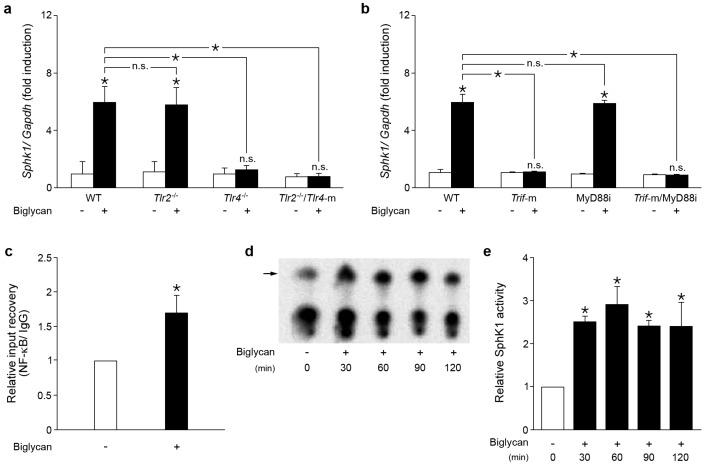
Biglycan triggers Sphk1 mRNA expression via TLR4/TRIF/NF-κB and stimulates SphK1 activity in macrophages. (**a**,**b**) qPCR analysis of Sphk1 normalized to Gapdh mRNA levels in biglycan-stimulated (4 μg/mL, 6 h): (**a**) WT, *Tlr2^−/−^*, *Tlr4^−/−^* and *Tlr2^−/−^/Tlr4*-m macrophages; and (**b**) WT and *Trif*-m macrophages pre-incubated with MyD88 inhibitor (50 μM, 30 min). (**c**) Chromatin immunoprecipitation with anti-NF-κB p65 antibody in WT macrophages stimulated with biglycan for 60 min followed by qPCR for Sphk1. The PCR primers bind at the transcription start site (TSS) or the promoter region. ChIP-qPCR analysis was normalized to IgG and given as fold induction of untreated control. (**d**) SphK activity assay based on sphingosine phosphorylation in the presence of [γ-^32^P] ATP in WT macrophages stimulated with biglycan for 30, 60, 90 and 120 min. Product separation was performed by TLC and detection with PharosFX Plus Molecular Imager (Bio-Rad, Munich, Germany). Arrow indicates [γ-^32^P] S1P. (**e**) Quantification of the resulting bands in (**d**). Data are expressed as means ± standard deviation (SD). (**a**,**b**) *n* = 5 individual experiments; (**c**,**d**,**e**) *n* = 3 individual experiments; * *p* < 0.05; n.s. = not significant. TLR: toll-like receptor; TRIF: Toll/IL-1R domain-containing adaptor inducing interferon (IFN)-β; NF-κB: nuclear factor κ-light-chain-enhancer of activated B-cells; SphK1: sphingosine kinase 1; qPCR: quantitative real-time polymerase chain reaction; ChIP: chromatin immunoprecipitation; WT: wild type; IgG: immunoglobulin G; MyD88: myeloid differentiation primary response protein; ATP: adenosine triphosphate; TLC: thin layer chromatography; S1P: sphingosine-1 phosphate; *Gapdh*: Glyceraldehyde 3-phosphate dehydrogenase.

To address the potential interference between biglycan and sphingolipid signaling in inflammation, thioglycolate-elicited primary macrophages isolated from wild-type (WT) C57BL/6 mice were stimulated with recombinant intact biglycan consisting of the protein core and two glycosaminoglycan side chains (4 μg/mL). After 30 min of incubation with biglycan already a significant increase in Sphk1 mRNA level was detected with time-dependent, consecutive Sphk1 expression enhancement up to five-fold after 6 h (Figure 1a, shown at 6 h of incubation).

Next, the receptor and adaptor molecules involved in biglycan-induced *Sphk1* expression were investigated, using WT, *Tlr2*^−/−^, *Tlr4*^−/−^ and *Tlr2*^−/−^/*Tlr4*-m macrophages. Quantitative real-time polymerase chain reaction (PCR) analysis revealed a similar level of Sphk1 mRNA in WT and *Tlr2*^−/−^ macrophages in response to biglycan, but no increase in *Tlr4*^−/−^ and *Tlr2*^−/−^/*Tlr4*-m macrophages (Figure 1a). Thus, biglycan signals through the TLR4 to induce Sphk1 mRNA expression.

To identify the TLR4 adaptor molecule involved in biglycan-dependent Sphk1 overexpression, macrophages from WT and *Trif*-m mice were pre-incubated for 30 min with the MyD88 inhibitor (50 μM) prior to stimulation with biglycan. Dysfunctional mutation of TRIF protein completely abolished biglycan induced Sphk1 expression, whereas the MyD88 inhibitor had no influence on its expression (Figure 1b). The functionality of the MyD88 inhibitor was proven by its inhibitory effects on biglycan-dependent induction of TNFα, as described previously [7]. To further demonstrate whether NF-κB is involved in biglycan/TLR4/TRIF induction of Sphk1, a chromatin immunoprecipitation (ChIP) assay was performed. Indeed, stimulation with biglycan (30–120 min) potentiated direct interaction between the NF-κB p65 subunit and the transcription start site (TSS) in WT macrophages (Figure 1c, shown at 60 min of incubation).

Next, we investigated whether biglycan is capable of inducing SphK1 activity in macrophages. In fact, 30–120 min of stimulation with biglycan resulted in a 2–3-fold enhancement of SphK1 activity in WT macrophages (Figure 1d,e). Collectively, we demonstrated that biglycan triggers via TLR4/TRIF/NF-κB the activity of *Sphk1* in murine macrophages.

### 2.2. Sphk2 Deficiency Potentiates Biglycan Triggered Sphk1 mRNA Expression

**Figure 2:**
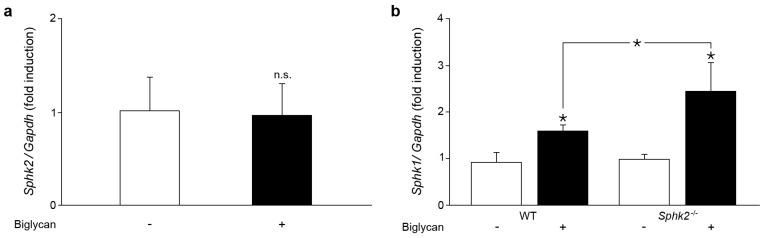
*Sphk2* deficiency potentiates biglycan-triggered Sphk1 mRNA expression. (**a**,**b**) qPCR analysis for mRNA levels of: (**a**) Sphk2 in WT macrophages stimulated with biglycan (4 μg/mL, 2 h); and (**b**) Sphk1 in WT and *Sphk2^−/−^* macrophages stimulated with biglycan (4 μg/mL, 2 h). mRNA expression was normalized to Gapdh and given as fold induction of untreated WT controls. Data are expressed as means ± SD. (**a**) *n* = 5 individual experiments; (**b**) *n* = 3 individual experiments; * *p* < 0.05; n.s. = not significant.

In the next set of experiments, the influence of biglycan on the expression of the sphingosine kinase isoform *Sphk2* was investigated. Biglycan had no effect on Sphk2 mRNA expression in WT macrophages during 30 min–6 h of incubation (Figure 2a, shown at 2 h of incubation).

In various cell types, deficient of *Sphk2*, a compensatory overexpression of Sphk1 mRNA has been reported [27,28]. Indeed, *Sphk2^−/−^* macrophages stimulated with biglycan for 2 h revealed a marked overexpression of Sphk1 mRNA (Figure 2b). Taken together, biglycan selectively upregulates Sphk1 expression in macrophages and this is more pronounced when SphK2 is lacking. Therefore, in the following experiments biglycan-stimulated *Sphk2^−/−^* macrophages were considered as Sphk1 overexpressing cells.

### 2.3. Biglycan Triggers CCL2 and CCL5 Production in a SphK1-Dependent Manner

**Figure 3:**
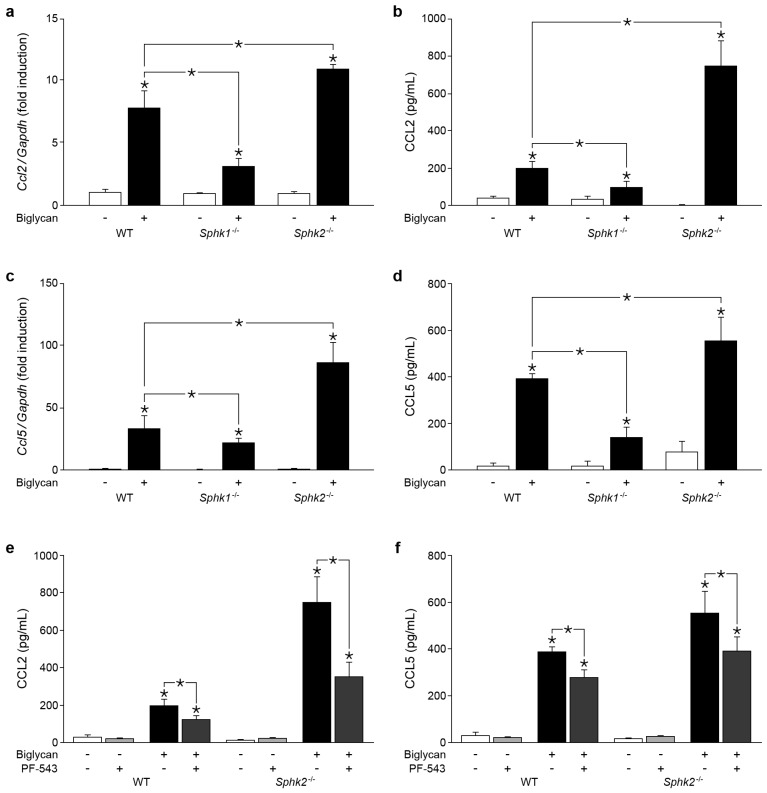
Biglycan triggers chemokine (C-C motif) ligand (CCL)2 and CCL5 production via SphK1 in macrophages. (**a**,**c**) qPCR analysis of mRNA levels of: (**a**) *Ccl2*; and (**c**) *Ccl5* in WT, *Sphk1^−/−^*, and *Sphk2^−/−^* macrophages stimulated with biglycan (4 μg/mL, 2 h). (**b**,**d**) Enzyme Linked Immunosorbent Assay (ELISA) for: CCL2 (**b**); and CCL5 (**d**) in the supernatants of WT, *Sphk1^−/−^*, and *Sphk2^−/−^* macrophages stimulated with biglycan (4 μg/mL, 2 h for CCL2 and 6 h for CCL5). (**e**,**f**) ELISA for: CCL2 (**e**); and CCL5 (**f**) in the supernatants of WT and *Sphk2^−/−^* macrophages stimulated with biglycan (4 μg/mL, 2 h for CCL2 and 6 h for CCL5) with and without the SphK1 inhibitors PF-543 (100 nM) applied 30 min prior to biglycan stimulation. mRNA expression was normalized to Gapdh and given as fold induction of untreated WT control. Data are expressed as means ± SD. (**a**–**f**) *n* = 3 individual experiments; * *p* < 0.05.

Previously, we have shown that biglycan triggers production of macrophage chemoattractants CCL2 and CCL5 [5,6,7,9]. As SphK1 modulates expression of various chemoattractants [23,29,30], we addressed the issue whether SphK is involved in biglycan-triggered production of CCL2 and CCL5. Indeed, *Sphk1* deficiency resulted in a marked reduction of biglycan-triggered Ccl2 mRNA expression in macrophages at 2 h of incubation (Figure 3a).

These data were further confirmed by decreased CCL2 protein abundance in cell supernatants from *Sphk1^−/−^* macrophages after 6 h of incubation with soluble biglycan (Figure 3b). By contrast, in *Sphk2^−/−^* macrophages overexpressing Sphk1 biglycan markedly induced Ccl2 mRNA expression (Figure 3a) resulting in enhanced CCL2 protein levels (Figure 3b). A similar pattern of biglycan/SphK1-dependent mRNA (Figure 3c) and protein (Figure 3d, shown at 6 h of incubation) regulation was obtained for CCL5.

To provide direct proof that biglycan-driven overexpression of CCL2 and CCL5 in cells lacking SphK2 is caused by SphK1, WT and *Sphk2^−/−^*, macrophages were incubated with biglycan in the presence of PF-543, a specific inhibitor of the SphK1 enzymatic activity [31,32]. As expected, this inhibitor reduced biglycan-dependent enhancement of CCL2 (Figure 3e) and CCL5 (Figure 3f) protein levels in supernatants from WT cells and abolished the chemokine overproduction in *Sphk2^−/−^* macrophages.

Thus, we provide here genetic and pharmacological proof that SphK1 is a crucial downstream mediator of biglycan-triggered CCL2 and CCL5 mRNA and protein expression in macrophages.

### 2.4. Biglycan Triggers Expression of Ccl2 via NF-κB, Erk1/2 and p38 MAPK, While Ccl5 Expression is Induced through NF-κB and p38 MAPK

**Figure 4:**
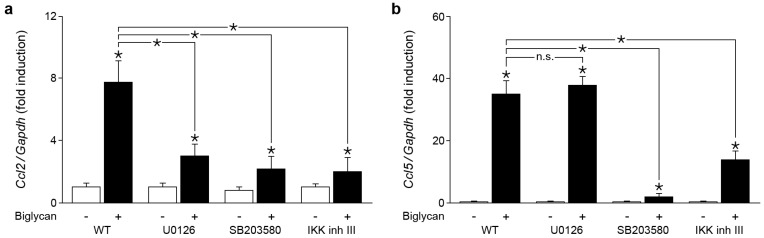
Biglycan triggers expression of Ccl2 and Ccl5 mRNA in Erk1/2-, p38 MAPK and NF-κB-dependent manner. (**a**,**b**) qPCR analysis for mRNA levels of: (**a**) Ccl2; and (**b**) Ccl5 in WT macrophages stimulated with biglycan (4 μg/mL, 2 h) with and without MEK/Erk inhibitor U0126 (10 μM), p38 MAPK inhibitor SB203580 (10 μM), and IKK inhibitor III (10 μM). The inhibitors were applied 30 min prior to the biglycan stimulation. mRNA expression was normalized to Gapdh and given as fold induction of untreated WT controls. Data are expressed as means ± SD. *n* = 3 individual experiments; * *p* < 0.05; n.s. = not significant. Erk1/2: extracellular signal-regulated kinase; p38 MAPK: p38 mitogen-activated protein kinase; IKK: IκB kinase; inh: inhibitor.

Our previous results demonstrated that biglycan induces the production of CCL2 in a TLR2/4/MyD88- and in the case of CCL5 in a TLR4/TRIF-dependent manner [6]. However, the gap in the signaling pathway between the adaptor and effector molecules had not been characterized. Therefore, we aimed to identify the kinase, which would be responsible for biglycan-, TLR2/4/MyD88-, and biglycan-TLR4/TRIF-triggered synthesis of CCL2 and CCL5, respectively. It is known that biglycan activates phosphorylation of Erk1/2, p38 MAPK and the translocation of NF-κB in macrophages [5]. Thus, we applied U0126, SB203580 and the IκB kinase (IKK) inhibitor III, the inhibitors of mitogen-activated protein kinase Erk kinase (MEK), p38 MAPK, and IκB kinase to WT macrophages for verification. Inhibition of IKK, Erk1/2 and p38 MAPK markedly reduced the biglycan-triggered Ccl2 mRNA expression (Figure 4a). In contrast, biglycan-dependent Ccl5 mRNA expression was reduced exclusively via IKK and p38 MAPK inhibitor (Figure 4b).

Thus, biglycan stimulates Ccl2 expression via TLR2/4/MyD88 and downstream activation of NF-κB, Erk1/2 and p38 MAPK, while Ccl5 expression is triggered by biglycan through TLR4/TRIF/NF-κB and p38 MAPK.

### 2.5. Control of Biglycan-Dependent Erk1/2, p38 MAPK and NF-κB Subunit p65 by SphK1 in Macrophages

**Figure 5:**
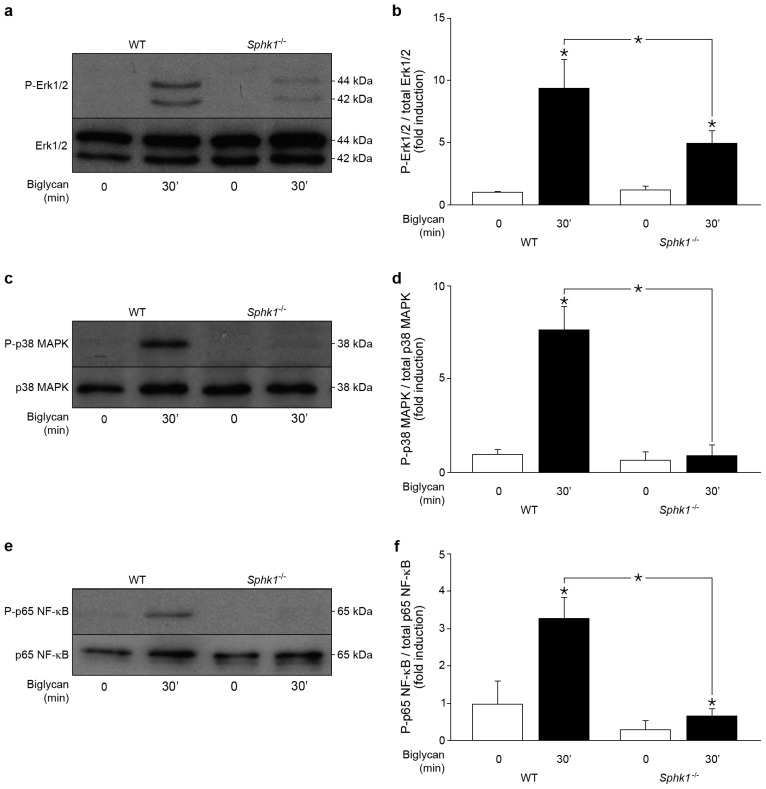
Biglycan phosphorylates Erk1/2, p38 MAPK and NF-κB p65 in SphK1-dependent manner in macrophages. (**a**,**c**,**e**) Western blot analysis of: (**a**) Erk1/2 phosphorylation; (**c**) p38 MAPK phosphorylation; and (**e**) NF-κB p65 phosphorylation in WT and *Sphk1^−/−^* macrophages stimulated with biglycan (4 μg/mL, 30 min). (**b**,**d**,**f**) Quantification of the resulting bands in (**a**,**c**,**e**), respectively, normalized to: (**a**) total Erk; (**c**) total p38; and (**e**) total p65. Data are expressed as means ± SD. *n* = 3 individual experiments; * *p* < 0.05.

As Erk1/2, p38 MAPKs, NF-κB, and SphK1 are mediators of the biglycan-triggered production of CCL2 and CCL5 chemokines, we evaluated whether SphK1 affects Erk1/2, p38 MAPK and NF-κB subunit p65 phosphorylation. In agreement with previous data, Western blot analysis demonstrated the phosphorylation of Erk1/2 (Figure 5a), p38 MAPK (Figure 5c) and p65 (Figure 5e) in WT macrophages incubated with biglycan for 30 min, which is a peak for biglycan stimulation of both Erk1/2 and p38 MAPK [5,7]. However, much less Erk1/2 (Figure 5a,b), p38 MAPK (Figure 5c,d) and p65 (Figure 5e,f) were found to be phosphorylated in biglycan-stimulated *Sphk1^−/−^* macrophages.

These data show that biglycan-induced phosphorylation of Erk1/2, p38 MAPK and p65 is SphK1-dependent. Thus, biglycan triggers CCL2 and CCL5 production in macrophages through SphK-controlled activation of Erk1/2, p38 MAPKs and NF-κB.

### 2.6. Soluble Biglycan Triggers Renal Expression of Ccl2 and Ccl5 and Macrophage Recruitment into the Kidney in Sphk1-Dependent Manner

**Figure 6:**
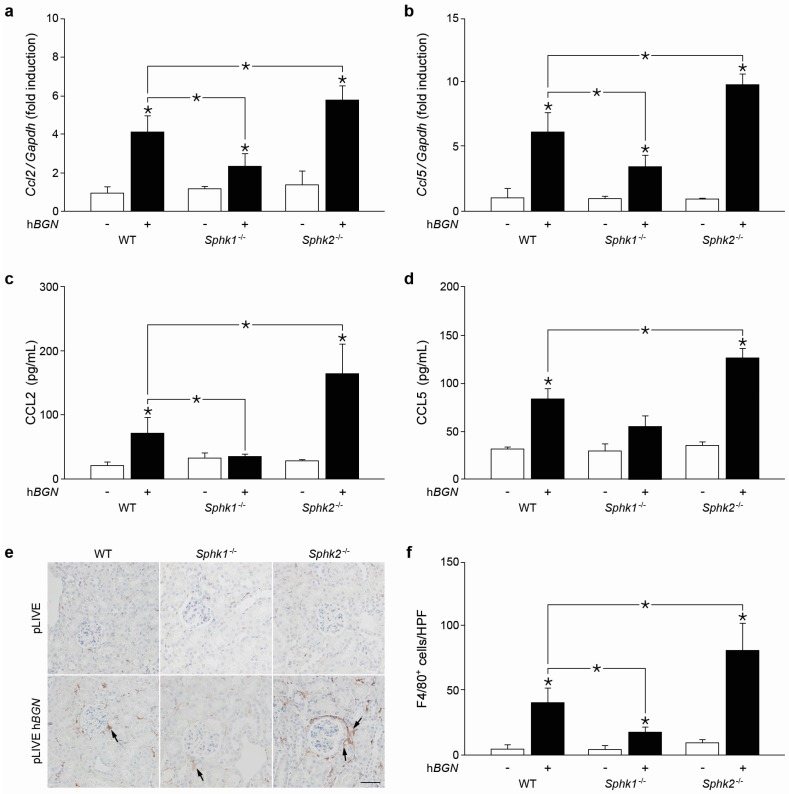
Biglycan induces Ccl2 and Ccl5 expression through SphK1 leading to renal recruitment of macrophages. (**a**,**b**) qPCR analysis of renal: (**a**) Ccl2; and (**b**) Ccl5 mRNA expression levels in pLIVE-h*BGN* and pLIVE (control) WT, *Sphk1^−/−^* and *Sphk2^−/−^* mice, three days of transfection. mRNA expression was normalized to Gapdh and given as fold induction of pLIVE-treated WT controls. (**c,d**) Plasma levels of: CCL2 (**c**); and CCL5 (**d**) in WT, *Sphk1^−/−^*, and *Sphk2^−/−^* mice, 3 days of transfection. (**e**) Immunohistochemical staining of the F4/80 macrophage marker (brown, depicted by arrows) in renal sections of WT, *Sphk1^−/−^*, and *Sphk2^−/−^* mice after injection of pLIVE-h*BGN* or pLIVE control, three days of transfection. Scale bar: 50 μm. (**f**) Quantification of F4/80^+^ cells given as cell count per high-power field (HPF). Data are expressed as means ± SD. *n* = 3 mice per group; * *p* < 0.05.

To address the in vivo relevance of these findings, human biglycan (pLIVE-h*BGN*) or empty pLIVE vector were transiently expressed (3 days) in WT, *Sphk1^−/−^* and *Sphk2^−/−^* murine livers under an albumin promoter [6,9]. Following transfection, soluble biglycan is released into the bloodstream and accumulates in various organs e.g., in the kidney [6,9]. Overexpression of human biglycan in the liver was confirmed by qPCR and restriction fragment length polymorphism analysis (data not shown) as described previously [6,9]. Plasma and renal levels of human biglycan were verified by Western blots [6,9]. As expected from our previous results, the Ccl2 (Figure 6a) and Ccl5 (Figure 6b) mRNA expression was elevated in WT pLIVE-h*BGN* vs. control pLIVE-kidneys [6].

Importantly, biglycan-dependent induction of renal Ccl2 (Figure 6a) and Ccl5 (Figure 6b) mRNA expression significantly declined in transfected pLIVE-h*BGN Sphk1*-deficient vs. pLIVE-h*BGN* WT mice. On the contrary, the expression of both chemokines was markedly enhanced in *Sphk2*-deficient pLIVE-h*BGN* as compared to pLIVE-h*BGN* WT kidneys (Figure 6a,b). This was associated with reduced plasma levels of CCL2 (Figure 6c) but not of CCL5 (Figure 6d) in pLIVE-h*BGN* *Sphk1^−/−^* mice and enhanced concentrations of circulating CCL2 (Figure 6c) and CCL5 (Figure 6d) in pLIVE-h*BGN* *Sphk2^−/−^* vs. WT transfected animals.

In addition, immunostaining for the macrophage marker F4/80 in renal sections from pLIVE-h*BGN*-injected mice revealed a lower number of macrophages in *Sphk1^−/−^* mice compared to WT (Figure 6e,f). On the other hand, enhanced macrophage infiltration was found in kidney sections from pLIVE-h*BGN*-transfected *Sphk2^−/−^* vs. WT kidneys (Figure 6e,f).

Thus, biglycan triggers expression of Ccl2 and Ccl5 and the recruitment of macrophages into the kidney in a *Sphk1*-dependent manner.

## 3. Discussion

**Figure 7:**
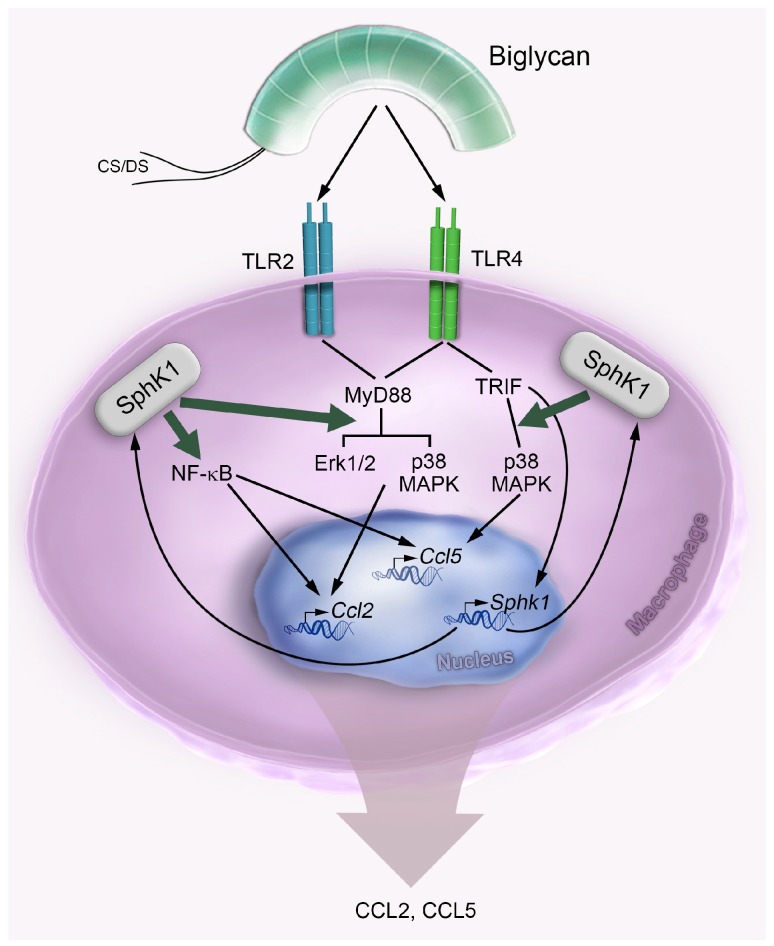
A working model summarizing the mechanisms of biglycan-driven and *Sphk1*-mediated production of macrophage chemoattractants CCL2 and CCL5. Following release from the ECM, biglycan interacts with TLR2 and -4 and triggers production of CCL2 and CCL5 through TLR2/4/MyD88 and TLR4/TRIF, respectively. By signaling via TLR4, biglycan induces the Sphk1 synthesis in a TLR4/TRIF-dependent manner. Moreover, biglycan induces the activity of SphK1. In turn, active SphK1 drives the biglycan-mediated production of CCL2 through Erk1/2 p38 MAPK and NF-κB activation, while CCL5 is induced only through p38 MAPK and NF-κB activation. Consequently, this leads to the recruitment of macrophages to inflamed tissues. Green arrows underline the effect of SphK1 on the biglycan-promoted NF-κB activation, MyD88/Erk1/2/p38 MAPK and TRIF/p38 MAPK pathways. Black arrows describe the biglycan-mediated inflammatory cascade.

The present report reveals sphingosine kinase SphK1 as a key contributor to the biglycan-driven inflammatory response in macrophages. Here we show that the ECM proteoglycan biglycan promotes the expression and activity of SphK1 through TLR4/TRIF/NF-κB and thus induces the production of the inflammatory chemoattractants CCL2 and CCL5 in macrophages. Biglycan triggers the synthesis and activity of SphK1 in a selective manner having no effect on the regulation of the SphK2 isoform. Mechanistically, biglycan potentiates the production of CCL2 via TLR2/TLR4/MyD88/Erk1/2/p38 MAPK/NF-κB and CCL5 via TLR4/TRIF/p38 MAPK/NF-κB in a SphK1-dependent manner, ultimately leading to the recruitment of macrophages into the kidney. The underlying mechanisms are graphically presented in Figure 7.

This is the first study showing that a component of the sphingolipid signaling network SphK1 is directly triggered by the ECM component biglycan. Diverse factors, such as TNFα [20,33,34], IL-1β [35], platelet-derived growth factor (PDGF) [36], transforming growth factor (TGFβ) [37,38], and nerve growth factor [39] have been reported to regulate SphK1. Among those factors, TGFβ and PDGF are not induced by biglycan [40,41]. By contrast, it is well known that biglycan acts as a trigger of TNFα and IL-1β protein in macrophages, requiring at least two hours of stimulation before the cytokines can be detected [5,9,37]. It is of note that biglycan-induced synthesis and activity of SphK1 as well as the interaction between NF-κB and *Sphk1* TSS occur already after 30 min. Therefore, it is conceivable that biglycan directly triggers SphK1 expression. At later time points, biglycan-induced TNFα and IL-1β might potentiate the direct effects of biglycan on SphK1 production. Hence, our data strongly suggest that biglycan directly induces SphK1 synthesis and activity.

Our findings regarding biglycan-dependent *Sphk1* induction are in agreement with several reports describing sphingosine kinases and sphingolipid metabolites to be involved in inflammatory reactions in response to various sterile danger signals or pathogens [17,33]. There are extensive studies, which show promoting effects of SphK1 on LPS- [20,23,42] and *Mycobacterium smegmatis*-triggered [43] expression of pro-inflammatory cytokines. Moreover, LPS cooperates with S1P to augment the expression of adhesion molecules and pro-inflammatory modulators [44]. S1P was shown to trigger cell death and NLR family pyrin domain containing 3 (NLRP3) inflammasome-dependent IL-1β secretion [30]. Additionally, sphingosine might act by itself as an endogenous DAMP [45].

Furthermore, we identified SphK1 as a crucial regulator of biglycan-dependent CCL2 and CCL5 production. Previously, we reported that soluble biglycan evokes CCL2 expression by engaging the TLR2/TLR4/MyD88 signaling pathway and CCL5 through TLR4/TRIF [6,7,9,11]. Here, we filled some of the signaling gaps between TLRs/ adaptor molecule complex and downstream cytokine synthesis. In macrophages genetically ablated or pharmacologically inhibited for SphK1, we discovered that SphK1 is a crucial mediator of biglycan-triggered Erk1/2, p38 MAPK, and NF-κB activation. Additionally, we found that biglycan triggers expression of Ccl2 through Erk1/2, p38 MAPK and NF-κB activation, while Ccl5 expression requires p38 MAPK and NF-κB. Importantly, SphK1 is a common upstream mediator of biglycan-dependent Erk1/2 p38 MAPK and NF-κB as well as of CCL2 and CCL5 synthesis. Thus, biglycan triggers the synthesis of SphK1 in macrophages in order to promote activation of Erk1/2, p38 MAPKs and NF-κB. Notably, this also represents a positive regulatory acceleration loop where NF-κB is required for SphK1 upregulation, which, in turn, triggers NF-κB activation. The activation of NF-κB by SphK1 is still controversially discussed. On one hand, it was shown that in mouse embryonic fibroblasts, SphK1 deficiency abolished TNFα-stimulated NF-κB activation [21], whereas, on the other hand, in macrophages of either *Sphk1* deficient or myeloid-specific *Sphk1*/*Sphk2* double deficient mice, no defect in TNFα- and LPS-induced inflammatory responses was detected, and these mice showed unaltered LPS-induced systemic inflammation and death [46].

Based on the literature and our data, it appears that SphK1 engages specific receptor/adapter molecule complexes, e.g., TLR4/MyD88 for LPS [47], TLR4/TRIF for biglycan, TLR2 for lipopeptides [48], and TNF receptor for TNFα [49] to stimulate the transcription of CCL2 and CCL5. Downstream of the receptors NF-κB [50] as well as various kinases such as Erk1/2 [23,51], protein kinase Cδ [34,52], phosphatidylinositol 3-kinase (PI3K) [23] or p38 MAPK were reported as mediators of CCL2 and CCL5 synthesis [43,44,51]. TNFα stimulates SphK1-mediated CCL5 through p38 MAPK [53], while *Mycobacterium* stimulates this cytokine through TLR2/SphK1 in a PI3K, Akt and IKKα/β-dependent fashion [54,55]. Taken in account a broad range of stimuli, receptors, adapter molecules, phosphorylation mediators as well as cell types, SphK1 appears to be a promising therapeutic target to regulate the macrophage CCL2 and CCL5 chemoattractants [56,57,58].

Our study has unveiled that biglycan selectively induces SphK1, whereas the sphingosine kinase isoform SphK2 expression remains unchanged upon biglycan stimulation. Additionally, biglycan-dependent SphK1 induction was more pronounced in *Sphk2*-deficient macrophages due to compensatory *Sphk1* upregulation. Consequently, higher CCL2 and CCL5 expression was detected in *Sphk2^−/−^* macrophages upon biglycan stimulation. Furthermore, the selective inhibitor of SphK1 activation [31] rescued the *Sphk1*-driven production of CCL2 and CCL5 in *Sphk2^−/−^* macrophages. This is in accordance with previous reports showing an inversed regulatory pattern of the *Sphk1* and *Sphk2* gens in various cell types and inflammatory disease models [24,53,59,60]. Furthermore, SphK1 but not SphK2-mediated S1P accelerates CCL2 expression in mast cells [61]. Thus, our findings provide strong evidence that biglycan selectively utilizes SphK1 to trigger CCL2 and CCL5 synthesis.

Importantly, we provided an in vivo proof of biglycan-SphK1-dependent CCL2 and CCL5 synthesis and macrophage recruitment. As previously described, transient overexpression of soluble biglycan [6,7,9,11] resulted in higher renal Ccl2 and Ccl5 expression as well as in enhanced numbers of infiltrating macrophages in the kidney [6,9]. Accordingly, the renal expression of both chemokines and the number of macrophages were markedly reduced in *Sphk1^−/−^* mice and abundant in kidneys lacking *Sphk2* vs. WT kidneys. Even though, there are no data addressing biglycan and sphingolipid interaction directly, SphKs and S1P have been studied in several renal diseases associated with overexpression of biglycan [11,62,63,64], namely diabetic nephropathy [65,66], glomerulonephritis [67], fibrosis [68], nephroblastoma [67] and acute kidney injury [69,70]. In this context, *Sphk1* deficiency increases albuminuria and glomerular connective tissue growth factor expression in diabetic nephropathy [66,70]. However, it was also reported that SphK1 deficiency results in CCL2 reduction and prevention of renal fibrosis in diabetic nephropathy [65]. In renal ischemia reperfusion injury, however, overexpression of *Sphk1* protects against inflammation and tubular damage [71]. Altogether, these data suggest that the effect of SphK1 on the outcome of renal inflammation is still controversial and appears to be disease- and duration-dependent [16]. Here, we report an anti-inflammatory effect of *Sphk1* deficiency in macrophage and kidney directly in a mouse model of transient overexpression of soluble biglycan.

In conclusion, we show for the first time that there is crosstalk between ECM- and sphingolipid-signaling. We have observed in vitro and in vivo evidence for biglycan-TLR4/TRIF-triggered SphK1 expression and activity. Our data provide new insights on how biglycan regulates CCL2 and CCL5 chemokines and macrophage recruitment into the kidney via SphK1. As SphK1 seems to impact on biglycan signaling upstream of Erk1/2, p38 MAPK and NF-κB, it is conceivable that SphK1 is a general regulator of various biglycan-triggered inflammatory responses.

## 4. Materials and Methods

### 4.1. Animal Experiments

Eight- to twelve-week-old male wild-type C57BL/6 mice were purchased from Charles River Laboratories (Sulzfeld, Germany). *Sphk1^−/−^* and *Sphk2^−/−^* mice have been described previously [28,65]. *Tlr2^−/−^* and *Tlr4^−/−^* mice were kindly provided by Marina Freudenberg (Max Planck Institute for Immunology, Freiburg, Germany). *Tlr2^−/−^/Tlr4*-m mice (*Tlr2^−/−^* mice carrying a TLR4 mutation) and *Trif*-m mice were a generous gift from Carsten Kirschning (Technical University of Munich, Munich, Germany) and Heinfried H. Radeke (University of Frankfurt, Frankfurt, Germany), respectively. All animal work was conducted in accordance with the German Animal Protection Law and was approved (F143/63, FU/K5591) by the Ethics Review Committee for laboratory animals of the District Government of Darmstadt, Germany.

### 4.2. In Vivo Transfection

Eight- to twelve-week-old wild-type C57BL/6, *Sphk1^−/−^* and *Sphk2^−/−^* male mice were anesthetized with 2% isoflurane (Abbott, Wiesbaden, Germany) under 1 L/min oxygen supply. For intravenous delivery, 50 μg of pLIVE-h*BGN* or pLIVE vector was incubated for 15 min before injection in sterile filtered 5% glucose containing 6 μl of Turbofect in vivo Transfection Reagent (Thermo Fisher Scientific, Darmstadt, Germany). The mice received a single intravenous injection and were sacrificed after 3 days of transfection. Plasma and liver were collected for analysis of h*BGN* overexpression. Kidneys were subjected to RNA extraction, Western blotting and histological analysis.

### 4.3. Immunohistochemistry

Sections (4 μm) of paraffin-embedded kidney samples from mice were blocked with 5% milk in Tris-buffered saline (TBS) with 0.05% Tween 20 for 1 h and incubated with the primary rat anti-mouse F4/80 (MCA497, Bio-Rad, AbDSerotec, Puchheim, Germany) antibody for 2 h at room temperature. The staining was developed with 3,3′-diaminobenzidine (Vector Laboratories, Peterborough, UK). Counterstaining was performed with Mayer’s Hematoxylin (AppliChem GmbH, Darmstadt, Germany). The specificity controls included omitting or replacement of primary antibody with rat unspecific IgG. The number of macrophages was estimated per high-power field (HPF 400×, with a minimum of 7 fields counted) (Soft Imaging System, Olympus, Münster, Germany). Histological examinations were performed by two observers blinded to the conditions.

### 4.4. Cell Culture and Stimulation

Thioglycolate-elicited macrophages were isolated from peritoneal lavage of wild-type C57BL/6, *Sphk1^−/−^*, *Sphk2^−/−^*, *Tlr2^−/−^*, *Tlr4^−/−^*, *Tlr2^−/−^/Tlr4*-m and *Trif*-m mice and grown in RPMI 1640 (Life Technologies, Darmstadt, Germany) supplemented with 1% penicillin and streptomycin and 2% fetal bovine serum (Biochrom, Berlin, Germany). Cells were stimulated with 4 μg/mL (80 nM) purified human biglycan [7] in serum-free medium for the indicated time points. For purification of the native proteoglycan, containing two chondroitin/dermatan sulfate chains, the conditioned medium of human biglycan-overexpressed 293 HEK cells was collected, passed over a DEAE-Trisacryl-M (Pall) column and further purified through high performance liquid chromatography [8]. The purity of the bigycan was verified by silver staining after sodium dodecyl sulfate (SDS) gel electrophoresis. MyD88 inhibitory peptide NBP2-29328 (50 μM; Novus Biologicals, Wiesbaden, Germany) was applied 30 min prior to stimulation with biglycan. SphK1 inhibitor PF-543 (100 nM, Cayman Chemical, Hamburg, Germany), MEK inhibitor U0126 (10 μM, Cell Signaling, Frankfurt am Main, Germany), p38 MAPK inhibitor SB203580 (10 μM, Calbiochem, Darmstadt, Germany) and IKK inhibitor III (10 μM, Merck, Darmstadt, Germany) were applied 30 min prior to stimulation with biglycan.

### 4.5. RNA Isolation and Quantitative Real-Time PCR

Total RNA was isolated using the TRI Reagent (Sigma Aldrich, Steinheim am Albuch, Germany) and was reverse transcribed using the High Capacity cDNA Reverse Transcription Kit (Applied Biosystems, Darmstadt, Germany). Real-time quantitative PCR was performed using AbiPrism 7500 Sequence Detection System (Applied Biosystem, Darmstadt, Germany). Quantitative RT-PCR was performed using TaqMan Fast Universal PCR Master Mix (Thermo Fisher Scientific, Darmstadt, Germany) and the following primers: *Ccl2* (Mm00441242_m1), *Ccl5* (Mm01302428_m1), *Gapdh* (Mm99999915_g1), *Sphk1* (Mm01252547_g1) and *Sphk2* (Mm00445021_m1). Relative changes in gene expression compared to control and normalized to Gapdh were quantified by the 2^−ΔΔ*C*t^ method.

### 4.6. Chromatin Immunoprecipitation

Preparation of cell extracts, crosslinking and isolation of nuclei was performed with the truCHIP^™^ Chromatin Shearing Kit (Covaris, Woburn, MA, USA) according to the manufacturers protocol. After sonification of the lysates with the Bioruptur Plus (10 cycles, 30 s on, 90 s off, 4 °C; Diagenode, Seraing, Belgium), cell debris was removed by centrifugation and the lysates were diluted 1:3 in dilution buffer (20 mmol/L Tris/HCl pH 7.4, 100 mmol/L NaCl, 2 mmol/L ethylenediaminetetraacetic acid (EDTA), 0.5% Triton X-100 and protease inhibitors). Pre-clearing was done with 20 µL DiaMag protein A coated magnetic beads slurry (Diagenode) for 45 min at 4 °C. The samples were incubated over night at 4 °C with the antibodies against NF-κB (3 µg Santa Cruz #sc-109, Hedelberg, Germany) or IgG (3 µg Diagenode # c15410206). Five percent of the samples served as input. The complexes were collected with 35 µL DiaMag protein A coated magnetic beads (Diagenode) for 3 h at 4 °C, subsequently washed twice for 5 min with each of the wash buffers 1–3 (Wash Buffer 1:20 mmol/L Tris/HCl pH 7.4, 150 mmol/L NaCl, 0.1% SDS, 2 mmol/L EDTA, 1% Triton X-100; Wash Buffer 2:20 mmol/L Tris/HCl pH 7.4, 500 mmol/L NaCl, 2 mmol/L EDTA, 1% Triton X-100; Wash Buffer 3:10 mmol/L Tris/HCl pH 7.4, 250 mmol/L lithium chloride, 1% Nonidet P-40, 1% sodium deoxycholate, 1 mmol/L EDTA) and finally washed with Tris-EDTA (TE)-buffer pH 8.0. Elution of the beads was done with elution buffer (0.1 M NaHCO3, 1% SDS) containing 1× Proteinase K (Diagenode) and shaking at 600 rpm for 1 h at 55 °C, 1 h at 62 °C and 10 min at 95 °C. After removal of the beads, the eluent was purified with the QiaQuick PCR purification kit (Qiagen, Hilden, Germany) and subjected to qPCR analysis. The following primer pairs for quantification of mouse *Sphk1* were used: forward primer 5′-TGA CGC GTG CGG AAC CGC AGG-3′ and reverse primer 5′-CTA TCT TCG CAT CGC TTC TTA AAG-3′ for the TSS of *Sphk1*.

### 4.7. Western Blot and ELISA

Total kidneys as well as macrophages were lysed in buffer containing 50 mM Tris/HCl (pH 8), 150 mM NaCl, 0.02% NaN_3_, 0.1% SDS, 1 μg/mL aprotinin, 1% Nonidet P-40, 0.5% sodium deoxycholate and protease inhibitors (100 μg/mL phenylmethylsulfonyl fluoride, 0.1 M ε-amino-n-caproic acid, 13 μM EDTA, 5 mM benzammoniumchloride monohydrate and 10 mM *N*-ethylmaleimide). For sodium dodecyl sulfate polyacrylamide gel electrophoresis (SDS-PAGE) and Western blotting, 50 μg of total protein from each sample were mixed with loading buffer (250 mM Tris/HCl pH 6.8, 8% sodium dodecyl sulfate, 40% glycerol, 8% β-mercaptoethanol, 0.02% Bromo phenol blue) and boiled at 95 °C for 5 min. Primary antibodies used were: mouse anti-β-actin (A5441, Sigma Aldrich, Steinheim am Albuch, Germany), rabbit anti-p38 MAPK (9212, Cell Signaling, Frankfurt am Main, Germany), rabbit anti-P-p38 MAPK (T180/Y182) (9211, Cell Signaling, Frankfurt am Main, Germany), rabbit anti-p44/42 MAPK (9102, Cell Signaling, Frankfurt am Main, Germany), rabbit anti-P-p44/42 MAPK (Thr202/Tyr204) (9101, Cell Signaling, Frankfurt am Main, Germany), rabbit anti-P-NF-κB p65 (Ser536) (93H1) (3033, Cell Signaling, Frankfurt am Main, Germany), and rabbit anti-NF-κB p65 (T180/Y182) (sc-109, Santa Cruz, Heidelberg, Germany). Secondary antibodies were HRP-coupled donkey anti-rabbit (NA934V, GE Healthcare, London, UK) and HRP-coupled sheep anti-mouse (NA931V, GE Healthcare, London, UK). Mouse CCL2/JE/MCP-1 and CCL5/RANTES DuoSet (R&D Systems, Wiesbaden, Germany) ELISA kit was employed and followed according to the manufacturer’s instructions.

### 4.8. Determination of Sphingosine Kinase Activity

Biglycan-stimulated primary murine macrophages were harvested and incubated with ice-cold 20 mM Tris buffer (pH 7.4) containing 20% glycerol, 1 mM β mercaptoethanol, 1 mM EDTA, phosphatase inhibitors (40 mM β-glycerophosphate, 1 mM sodium orthovanadate, and 15 mM sodium fluoride), protease inhibitors (10 μg/mL leupeptin, 10 μg/mL aprotinin, and 1 mM phenylmethylsulfonyl fluoride). The supernatant was assayed for sphingosine kinase activity by incubation with sphingosine (Sigma) and [γ-^32^P] ATP for 15 min at 37 °C. The products were separated on a thin layer chromatography (TLC) plate using 1-butanol/ethanol/acetic acid/water (80:20:10:20) and visualized on PharosFX Plus Molecular Imager (Bio-Rad, Munich, Germany).

### 4.9. Statistics

All data are expressed as means ± standard deviation (SD). Two-sided Student’s *t*-test was used to evaluate significance of differences between groups. Differences were considered significant at *p* < 0.05.

## Abbreviations

CCL	chemokine (C-C motif) ligand
CXCL	chemokine (C-X-C motif) ligand
DAMP	damage-associated molecular patterns
ECM	extracellular matrix
IL-1	interleukin-1
LPS	lipopolysaccharide
MAPK	mitogen-activated protein kinase
MyD88	myeloid differentiation primary response protein
NF-κB	nuclear factor κ-light-chain-enhancer of activated B-cells
S1P	sphingosine-1 phosphate
SphK	sphingosine kinase
TLR	Toll-like receptor
TNF	tumor necrosis factor
TRIF	Toll/IL-1R domain-containing adaptor inducing IFN-β
TSS	transcription start site
WT	wild-type
